# Beta-blocking agents in cardiovascular disease; are they here to stay?

**DOI:** 10.1007/s12471-014-0588-y

**Published:** 2014-08-20

**Authors:** E. E. van der Wall

**Affiliations:** Interuniversity Cardiology Institute of the Netherlands (ICIN) - Netherlands Heart Institute (NHI), P.O. Box 19258, 3501 DG Utrecht, the Netherlands

In the contemporary practice of treatment of myocardial infarction (MI), beta-blocking agents have no mortality benefit but they do reduce recurrent myocardial infarction (MI) and angina. However, this comes at the expense of an increase in heart failure, cardiogenic shock and drug discontinuation as shown by a recent meta-analysis published online in the American Journal of Medicine in June 2014 [1]. This meta-analysis involved 60 trials with 102,003 patients and the primary outcome was all-cause mortality. The authors concluded that clinical guidelines recommending the use of beta-blocking agents in post-MI patients need to be reconsidered. Consequently, debate has arisen regarding the efficacy of beta-blocking agents in MI and in other cardiovascular conditions.

Let’s review some recent trials on the application of beta-blocking agents in patients with various cardiovascular diseases and under various circumstances.

## Beta-blocking agents in angina and MI

The role of beta-blockers in patients with coronary risk factors or a remote MI or stroke has been assessed in a meta-analysis, regrouping 44,708 patients from the Reduction of Atherothrombosis for Continued Health (REACH) Registry [2]. In this observational study of patients with either risk factors only, known prior MI, or known coronary artery disease (CAD) without MI, the use of beta-blockers was not associated with a lower risk of composite cardiovascular events after a 44-month median follow-up. The authors clearly pointed to the fact that the role of beta-blocking agents for secondary prevention in these patients should be reconsidered.

## Beta-blocking agents and percutaneous coronary intervention (PCI)

Osaza et al. [3] studied the influence of beta-blocker therapy on prognosis in 5288 patients with CAD who underwent percutaneous coronary intervention (PCI). The patients had no history of MI or heart failure. Beta-blocker therapy was associated with worse 3-year clinical outcomes in CAD patients who underwent PCI. As a result, randomised trials are warranted to identify appropriate subsets of patients who could truly benefit from long-term use of beta-blockers in this setting.

## Beta-blocking agents and heart failure

Patients with heart failure in whom there is no contraindication should receive beta-blocker therapy. This pertains currently to 120,000 patients in the Netherlands raising to 200,000 in the coming decade [4]. Results from major studies involving bisoprolol, carvedilol, and metoprolol have demonstrated significant morbidity and mortality benefits, with a mortality reduction of approximately 35 % across trials. The Study of the Effects of Nebivolol Intervention on Outcomes and Rehospitalisation in Seniors with Heart Failure (SENIORS) in 2128 patients aged 70 years and older with heart failure independent of left ventricular ejection fraction at entry demonstrated that nebivolol significantly reduced the composite outcome of all-cause mortality and cardiovascular hospital admission by 14 %; however, nebivolol did not reduce the risk of all-cause mortality compared with placebo [5]. Overall, the efficacy and tolerability of bisoprolol, carvedilol and metoprolol are similar in patients with systolic heart failure, but nebivolol is not well-tolerated and is less effective than the other beta-blocking agents possibly due to less reduction in heart rate [6, 7]. Importantly, in patients with unstable severe heart failure, substantial evidence for the use of beta-blocking agents is lacking. In these patients temporary lowering or discontinuation of beta-blocker therapy may be necessary and lifesaving [8].

## Beta-blocking agents and hypertension

Beta-adrenergic receptor blockers have been used for more than 45 years in the treatment of hypertension. Recent meta-analyses, which specifically addressed the effect of beta-blockers in the treatment of hypertension, have confirmed their efficacy in the reduction of cardiovascular morbidity and mortality [9, 10]. However, the beneficial effect was mainly seen in younger patients (<60 years) and less in older patients. In the elderly population there is an excess risk of stroke with beta-blockers compared with other antihypertensive agents. This class of agents decreases cardiovascular endpoints in younger patients, suggesting that age might be a more important factor than the choice of the beta-blocking agent.

## Beta-blocking agents and coronary artery bypass grafting

Use of preoperative beta-blockers has been associated with a reduction in perioperative mortality for patients undergoing coronary artery bypass grafting (CABG) in observational research studies. This has led to the adoption of preoperative beta-blocker therapy as a quality standard. However, Brinkman et al. [11] recently showed in a retrospective analysis of the Society of Thoracic Surgeons National Adult Cardiac database for 1107 hospitals performing CABG in the US in 506,110 patients from 2008 to 2012, that preoperative beta-blocker use among patients undergoing CABG surgery was not associated with improved perioperative outcomes.

## Beta-blocking agents and non-cardiac surgery

The cardiovascular benefit of perioperative beta-blocker therapy for patients undergoing non-cardiac surgery has been the subject of debate for more than two decades. In recent years, the controversy has been renewed by conflicting results of studies that have shown both protective and harmful effects [12]. A recent meta-analysis by Bouri et al. [13] of nine trials including 10,529 patients demonstrated an increased risk of 30-day mortality among patients receiving a beta-blocking agent. It has been proclaimed that using beta-blocking agents in non-cardiac surgery - a policy previously advocated by the European Society of Cardiology (ESC) guideline - might have caused 10,000 iatrogenic deaths each year in the UK [14]. In reality it is of course not known how universally the guideline is followed, so the actual number of presumed deaths is probably much smaller.

### New guidelines on non-cardiac surgery!

The inherent ESC guideline –endorsed by our national society, the NVVC- has recently been adapted according to current knowledge and has been published, jointly with the European Society of Anaesthesiology (ESA), under the title Non-Cardiac Surgery: Cardiovascular Assessment & Management (Chairpersons Steen Dalby Kristensen, Juhani Knuuti & Stefan De Hert). The updated 112-page document with 279 references is freely downloadable from the ESC and European Heart Journal (EHJ) websites and was published online in the EHJ on 1 August 2014 [15]. The 2014 ESC/ESA Guidelines cover the entire field including surgical risk assessment, preoperative evaluation, and optimal perioperative management, and also address relevant cardiology and anaesthesiology issues in patients with specific cardiac diseases and common comorbidities who are scheduled to undergo non-cardiac surgery. The new 2014 ESC/ESA Guidelines state that beta-blockers are no longer recommended in patients scheduled for low or intermediate risk surgery. The initiation of beta-blockers in patients who undergo non-cardiac surgery should not be considered routine. With most patients, the use of beta-blockers should be evaluated by their clinician unless specifically stated otherwise. Perioperative continuation of beta-blockers is recommended in patients currently treated with beta-blockers. Preoperative initiation of beta-blockers may be considered in patients scheduled for high-risk surgery and who have ≥2 clinical risk factors or American Society of Anesthesiologists (ASA) status ≥3 and those who have known ischaemic heart disease or myocardial ischaemia. Nevertheless, initiation of perioperative high-dose beta-blockers without titration is not recommended. These 2014 Guidelines were released simultaneously with the American College of Cardiology/American Heart Association Guidelines on the same topic, published in both the Journal of the American College of Cardiology (JACC) and Circulation.

To summarise, over the past years beta-blocking agents have gained a fixed niche in the pharmaceutical management of patients with a variety of cardiovascular diseases. It speaks for itself that they are here to stay. However, recent clinical trials have been rather sceptical about the generally accepted positive effects of these drugs in cardiovascular medicine independent of the nature of the disease. Consequently, the indiscriminate use of beta-blocker therapy as a panacea for cardiovascular diseases in general should be highly discouraged and newly developed guidelines should reconsider its appropriate use and be modified accordingly as has recently been demonstrated for the use of beta-blocking agents in patients undergoing non-cardiac surgery.
